# The Prognostic and Predictive Value of Genomic Assays in Guiding Adjuvant Breast Radiation Therapy

**DOI:** 10.3390/biomedicines11010098

**Published:** 2022-12-30

**Authors:** Sasha J. Beyer, Miranda Tallman, Sachin R. Jhawar, Julia R. White, Jose G. Bazan

**Affiliations:** 1 Department of Radiation Oncology, The Ohio State University Wexner Medical Center, Columbus, OH 43210, USA; 2 Department of Radiation Oncology, The University of Kansas Medical Center, Kansas City, KS 66160, USA; 3 Department of Radiation Oncology, City of Hope Comprehensive Cancer Center, Duarte, CA 91010, USA

**Keywords:** breast cancer, radiation therapy, genomic assays

## Abstract

Many patients with non-metastatic breast cancer benefit from adjuvant radiation therapy after lumpectomy or mastectomy on the basis of many randomized trials. However, there are many patients that have such low risks of recurrence after surgery that de-intensification of therapy by either reducing the treatment volume or omitting radiation altogether may be appropriate options. On the other hand, dose intensification may be necessary for more aggressive breast cancers. Until recently, these treatment decisions were based solely on clinicopathologic factors. Here, we review the current literature on the role of genomic assays as prognostic and/or predictive biomarkers to help guide adjuvant radiation therapy decision-making.

## 1. Breast Cancer and Breast Conservation Therapy

Breast conservation therapy (BCT) consisting of a lumpectomy followed by radiation therapy was first shown in the 1980’s to have equivalent outcomes to mastectomy for early stage invasive breast cancers [1,2,3,4,5,6]. Breast radiation after breast conservation surgery improves local recurrence rates and overall survival at 15 years [1,2]. BCT has therefore become a standard treatment paradigm for treating invasive breast cancers [1,7,8]. Current treatment guidelines recommend radiation therapy for most patients having breast conservation surgery.

While adjuvant radiation has therapeutic advantages, daily radiation treatments can be inconvenient, costly and may involve toxicity to the heart, lungs, skin and adverse cosmesis [7,8,9]. For these reasons, there has been a trend toward decreasing the burden and inconvenience of daily radiation treatments among low-risk breast cancer patients (defined as post-menopausal women with lymph node negative, estrogen receptor (ER) positive cancers of small size < 2 cm and negative margins). Multiple large randomized studies have shown that reducing radiation treatment volumes with accelerated partial breast irradiation (APBI) [10,11,12,13,14] or reducing overall treatment times with an ultrahypofractionated approach [15,16] does not compromise cancer control compared to conventional whole breast radiation in select low risk breast cancer subpopulations.

The identification of low risk breast cancers in which radiation may be safely omitted and the side effects of radiation can be spared is an area of investigation. Historical clinical trials omitting radiation therapy for low risk breast cancers based on clinical and pathologic factors alone have shown conflicting results. More recently, prognostic genomic assays have shown promise in improving our ability to identify patients with a low risk of local recurrence in which adjuvant radiation may be safely omitted to prevent radiation-associated toxicities. Prospective clinical trials to investigate the ability of prognostic genomic assays to identify low risk breast cancers in which radiation can be safely omitted are currently ongoing. While prognostic genomic assays may help stratify breast cancers according to their risk of recurrence, the development and validation of predictive genomic assays may help us to better understand the likelihood of breast cancers to respond to radiation therapy, thus allowing for a more tailored locoregional approach to improve radiation outcomes. In this review, we summarize the current evidence for prognostic genomic assays and discuss the potential for predictive genomic assays to facilitate adjuvant radiation therapy decisions in the clinic.

## 2. De-Escalation of Radiation Therapy Based on Clinical Factors in Breast Cancer

The historical NSABP-06 trial randomized a non-selective cohort of *n* = 1851 early stage breast cancer patients to lumpectomy, lumpectomy and radiation, or mastectomy. The 20-year outcomes showed the incidence of recurrent tumor in the ipsilateral breast was 14.3% in women undergoing lumpectomy followed by breast irradiation compared to 39.2% in the women undergoing lumpectomy alone (*p* < 0.001) [4]. This study, which included all subtypes of early stage invasive breast cancers, indicated that radiation after lumpectomy substantially improved the rates of local recurrence, thus confirming lumpectomy followed by radiation as standard therapy for early stage breast cancers. Further retrospective studies have also suggested that omission of whole breast radiation post-lumpectomy in higher risk early stage breast cancers may decrease survival [17,18].

Several clinical studies have made attempts to identify “low risk” ER+ early stage breast cancers in which radiation can be omitted with similar rates of local control. NSABP-21 evaluated whether radiation could safely be omitted in select early stage breast cancer patients with small ER+ or ER− tumors < 1 cm and negative surgical margins [19]. In this study, *n* = 1009 patients with T1a or T1b breast cancers were randomized to tamoxifen alone, radiation + placebo, or radiation + tamoxifen. At 8 years, the cumulative incidence of in breast tumor recurrence was 16.5% with tamoxifen alone, 9.3% with radiation and placebo, and 2.8% with radiation and tamoxifen. Thus, radiation reduced local recurrence below the level achieved with tamoxifen alone [19]. Similar results were shown in the Austrian Breast and Colorectal Cancer Study Group (ABCSG) trial 8A, a prospective, multicenter clinical trial that randomized favorable early stage breast cancer patients (<3 cm, Grade 1 or 2, ER/PR+) receiving lumpectomy with radiation versus lumpectomy without radiation [20]. The 5-year recurrence rates were 2.1% in the radiation group versus 6.1% in the no-radiation group (*p* = 0.002) [20]. The results of the Swedish Breast Cancer Group 91 RT (SweBCG 91 RT) also confirmed that small early stage invasive breast cancers identified on screening mammogram had an improved in-breast tumor recurrence benefit from radiation therapy (23.9% vs. 11.5%, *p* < 00.1) [21,22].

While these randomized trials demonstrated a local control benefit of radiation in ER+ early stage breast cancers, elderly women with small, low grade ER+ breast cancers appeared to derive the least benefit [23]. In the Cancer and Leukemia Group B (CALGB) 9343 trial, 636 women aged ≥ 70 years old with T1 early stage ER+ breast cancers were randomized to receive tamoxifen alone versus tamoxifen plus radiation therapy post-lumpectomy [24,25]. At a long term follow up of 12.6 years, the local recurrence rates were only mildly improved with radiation (10% in the tamoxifen alone group vs. 2% in the tamoxifen and radiation group). However, the secondary endpoints of overall survival, distant metastases and mastectomy-free survival were the same between the two groups [24]. The PRIME-II trial, which included ER+ early stage breast cancer patients ≥ 65 years old with T1/T2 tumors demonstrated similar results with only mildly improved rates of local recurrence with radiation post-lumpectomy (4.1% vs. 1.3%) [26]. Given only a modest local control benefit of radiation, NCCN guidelines were revised to allow for the omission of radiation in patients ≥ 70 yo in 2005 [27].

In 2004, Fyles et al. [28] randomized *n* = 769 women ≥ 50 yo with T1/T2 early stage ER+, node-negative breast cancers to tamoxifen alone compared to tamoxifen plus radiation. Despite this favorable early stage ER+ breast cancer patient population, radiation significantly reduced the rate of local relapse. After a median follow up of 5.6 years, the risk of local relapse was 7.7% in the tamoxifen alone group compared to only 0.6% in the radiation plus tamoxifen group [28]. However, a further retrospective analysis by Liu et al. [29], showed a minimal rate of recurrence regardless of radiation in a subtype of breast cancer defined as ER+/PR+/Her-2 negative with a low Ki-67 value. These results prompted the question of whether better characterizing the biology of breast cancer may help identify breast cancers in which radiation has minimal benefit and can be safely omitted.

Most recently, the LUMINA single arm prospective clinical trial [30] showed that women ≥ 55 yo with Grade 1–2 invasive breast cancer of the Luminal A subtype (defined as: ER ≥ 1%, PR > 20%, HER2 negative and Ki67 ≤ 13.25%) had very low rates of local recurrence of 2.3% at 5 years when patients were treated with lumpectomy followed by endocrine therapy alone [31]. While these preliminary results show promise that Luminal A breast cancers may be candidates for radiation omission, the results of multiple prospective clinical trials to further characterize the role of adjuvant radiation among breast cancer molecular subtypes using genomic assays are ongoing.

## 3. Genomic Profiling of Breast Cancers

It is well-known that breast cancers represent a heterogenous group of diseases composed of several subtypes. Seminal genomic profiling studies have shown that breast cancer is composed of intrinsic molecular subtypes [32,33,34,35]. The Luminal A, Luminal B, Her-2 enriched and Basal-like molecular subtypes demonstrate different disease-specific outcomes and treatment responses. Multiple studies have shown that these molecular subtypes of breast cancer strongly predict locoregional recurrence in addition to distant recurrence [36,37,38,39,40]. While molecular subtypes facilitate adjuvant systemic and radiation treatment decisions, the use of genome-wide gene expression profiling microarrays can be costly, time consuming and challenging to implement in the clinic.

Consequently, molecular subtypes are often approximated in the clinic by immunohistochemical staining for ER, PR and Her-2 status, grade and Ki-67 proliferation rate. Multiple studies have shown that Luminal A breast cancers had the lowest rate of regional local recurrence and best prognosis. In contrast, Her-2 enriched and Basal-like subtypes demonstrated an increased risk of local recurrence [37,38,39,40]. Arvold et al. [37] approximated these subtypes based on immunohistochemistry and grade and determined the 5-year cumulative incidence of local recurrence was 0.8% for Luminal A, 2.3% for Luminal B, 1.1% for Luminal HER-2, 10.8% for HER2-enriched and 6.7% for Basal-like early stage breast cancers. Moreover, Voduc et al. [39] defined these molecular subtypes based on a six gene panel, including ER, PR, Her-2, Ki-67, epidermal growth factor receptor (EGFR), and cytokeratin (CK) 5/6. Bane et al. [38] classified tumors by molecular subtype, including Luminal A, Luminal B, Her-2 enriched, Basal-like or unclassified based on immunohistochemical staining for ER, PR, Her-2, Ki-67, CK 5/6, EGFR status in addition to HIF-1alpha, CAIX and GLUT1. In this study, the 10 yr cumulative local recurrence incidence was 4.5% for Luminal A and Basal-like, 7.9% for Luminal B and 16.9% for Her-2 enriched tumors [38].

Commercially available genomic assays have now been developed and are commonly implemented in the clinic as prognostic and predictive tools for adjuvant systemic therapy. In the next section, we discuss OncotypeDx, Mammoprint, and other commercially available gene assays for guiding adjuvant systemic therapy decisions in the clinic.

## 4. Genome Expression Assays as Prognostic and Predictive Biomarkers for Adjuvant Systemic Therapy

Genomic assays are now routinely used for making adjuvant systemic treatment decisions in the clinic [41,42,43,44,45,46]. OncotypeDx (Exact Sciences, Madison, WI, USA) is a multigene assay using RNA expression profiling on a panel of 21 genes, including 16 cancer-related genes associated with proliferation, invasion, and estrogen signaling in addition to 5 reference genes [47]. Gene expression levels are included in a mathematical algorithm to generate a Recurrence Score (RS) from 0–100 that stratifies patients into three different risk categories of low (0–18), intermediate (18–31), and high (31–100) risk of distant recurrence.

Multiple retrospective studies showed that Recurrence Scores using this 21 gene assay could not only estimate the risk of distant recurrence in ER+, node-negative breast cancers on NSABP B-14 and NSABP B-20 [48,49,50,51,52], however it could also predict breast cancers most likely to benefit from adjuvant chemotherapy [49]. Indeed, Paik et al. [49] showed that patients with high Recurrence Scores (≥31) experienced a large benefit from cyclophosphamide, methotrexate, and fluorouracil chemotherapy (27.6% absolute mean decrease in distant recurrence rate at 10 years), while patients that had low Recurrence Scores (<18) experienced minimal benefit from chemotherapy (1.1% absolute mean decrease in distant recurrence at 10 years). While the role for chemotherapy was clear in high and low risk cases, it was less certain in patients with intermediate Recurrence Scores [49].

The Trial Assigning Individualized Options for Treatment (TAILORx) was a prospective randomized clinical trial designed to address this uncertainty in chemotherapy benefit among intermediate Recurrence Scores [53]. ER+, node-negative breast cancer patients with intermediate (11–25) Recurrence Scores were randomized to receive either chemotherapy and endocrine therapy or endocrine therapy alone [53]. Patients with Recurrence Scores between 11 and 25 had similar outcomes whether treated with chemoendocrine therapy or endocrine therapy alone. Both groups had a disease-free survival rate of ~84%, disease-free metastasis of ~95% and overall survival of ~93% at a follow up of 9 years. However, some survival benefit was observed in women < 50 yo with Recurrence Scores of 16 to 25, with benefit increasing as the Recurrence Score increased [53,54].

In regard to node positive patients, Albain et al. [55] performed a retrospective analysis that showed the 21-gene Recurrence Score to also be prognostic and predictive of chemotherapy benefit in a subset of post-menopausal, ER+, node positive women on the Southwest Oncology Group (SWOG)-8814 INT-0100 trial [55,56]. In this study, patients with Recurrence Scores < 18 showed no benefit to chemotherapy, however patients with Recurrence Scores ≥ 31 showed increased disease-free survival, overall survival and breast cancer specific survival in patients treated with chemotherapy [55]. The prognostic and predictive role of Recurrence Scores in node-positive breast cancers was evaluated by RxPonder (A Clinical Trial RX for Positive Node, Endocrine Responsive Breast Cancer) [57], a prospective clinical trial randomizing women with ER+, node positive (1–3 positive lymph nodes) and Recurrence Scores of 0–25 to chemotherapy plus endocrine therapy vs. endocrine therapy alone. The RxPonder trials showed that ER+ post-menopausal women with 1–3 positive lymph nodes and a recurrence score of 0–25 showed no clinically relevant benefit to adjuvant chemotherapy. However, for the 33% of pre-menopausal women enrolled on the trial, an increase in disease-free survival of 40% and relapse-free survival of 42% was seen with the addition of chemotherapy regardless of recurrence score [57]. As a result, genomic assays are used in making adjuvant systemic therapy decisions for post-menopausal women, but not pre-menopausal women, with 1–3 positive nodes.

MammaPrint (Agendia Precision Oncology, Irvine, CA, USA) is a prognostic 70-gene signature developed by the Netherlands Cancer Institute that out-performed all clinical variables in predicting distant metastases over five years. The 70 gene signature involving genes associated with cell cycle, invasion, metastases and signal transduction was able to classify breast cancer patients with tumor size < 5 cm, lymph node negative and age < 55 yo into “poor prognosis” and “good prognosis” subtypes [58,59]. The initial validation study showed that node-negative breast cancer patients stratified as low risk by the 70-gene signature demonstrated a 10-year overall survival of 92%, whereas patients classified as high risk had a 59.5% 10-year overall survival [60]. The prospective Phase III MINDACT (Microarray In Node-Negative and 1 to 3 Positive Lymph Node Disease May Avoid Chemotherapy Trial) later showed that the 70 gene signature identified patients with high clinical risk and low genomic risk of distant metastases that did not benefit from the addition of chemotherapy to endocrine therapy at a median follow up of 8.7 years [61,62]. More recent studies suggest that this 70-gene Mammaprint signature may identify ultra-low risk breast cancers that could be candidates for further de-escalation of endocrine therapy [63,64].

Based on these previously discussed multiple prospective clinical trials, ASCO guidelines provide evidence-based recommendations for the use of genomic assays for guiding adjuvant systemic therapies among early-stage breast cancers [43,45,46].

## 5. Prognostic Genomic Assays and Adjuvant Radiation Therapy

While the past decade has seen a significant increase in the utilization of genomic assays in making adjuvant systemic therapy recommendations, the relevance of genomic assays in guiding radiation therapy recommendations remains uncertain. Clinicopathologic factors such as grade, tumor size, lymph node status and age independently correlate with local recurrence. However, prospective clinical trials aimed to identify low-risk breast cancers based on clinicopathologic factors alone in which radiation therapy can be omitted with minimal effect on disease control have been unsuccessful. Radiation provided at least a modest benefit in local control in all the studies [19,20,24,25,26,65].

The previously described Oncotype and Mammaprint assays are not only prognostic for distant recurrence, but have also been shown to be prognostic for local recurrence [50,66,67,68,69,70]. In 895 tamoxifen-treated ER+, node negative patients from NSABP B-14 and B-20, locoregional recurrence (LRR) was significantly associated with Recurrence Score. The 10-year LRR was 4.3% for patients with a low RS (<18), 7.2% for those with intermediate RS (18–30), and 15.8% for those with a high RS (≥30) [50]. Recurrence Score remained an independent predictor of LRR in ER+, node-positive patients treated with adjuvant chemotherapy plus tamoxifen in NSABP B-28 [71].

The risk-of-recurrence (ROR) score derived from the Predictor Analysis of Microarray (PAM) 50 (Prosigna) is a 50 gene assay that describes individual breast cancers based on intrinsic subtype and has also been shown to be an independent prognostic factor for risk of local recurrence in post-menopausal ER+ early stage breast cancer patients [72,73,74]. Women with breast cancer who had a low-risk PAM-50 ROR score (defined as <57) after breast conservation therapy on the Austrian Breast and Colorectal Cancer Study Group (ABCSG) 8 randomized trial had only a 0.9% risk of local recurrence [72]. These prognostic genomic assays have been summarized in Table 1.

Current prospective clinical trials are evaluating whether OncotypeDx and PAM-50 prognostic gene assays can be used to identify low risk ER+ early breast cancer patients in which radiation therapy can be safely omitted (Table 2). The IDEA (Individualized Decisions for Endocrine Therapy Alone) is a single arm Phase II prospective trial evaluating locoregional recurrence rates in low risk women (defined as post-menopausal, ER+ with a RS ≤ 18 who plan to receive endocrine therapy) in which radiation therapy will be omitted after breast conservation surgery [75]. The PRECISION (Profiling Early Breast Cancer for Radiation Omission) is a prospective Phase II single arm trial evaluating 5 year locoregional recurrence after the omission of radiation ER+ early stage breast cancers < 2 cm with a low risk PAM-50-based ROR in post-menopausal woman > 50 yo [76]. Both the IDEA and PRECISION trials have completed accrual. NRG-BR007 DEBRA (De-Escalation of Breast Radiation) is a currently accruing Phase III prospective, randomized trial evaluating omission of breast radiation in ER+/Her-2 negative early stage breast cancers with Oncotype Recurrence Scores ≤ 18 in women age 50–69 yo [77]. The TAILOR-RT Phase III is randomizing patients to regional nodal radiation that have ER+/Her2-negative biomarker low risk (defined as Oncotype ≤ 25) breast cancers with low lymph node burden (1–3+ lymph nodes) [78]. The EXPERT (Examining Personalized Radiation Therapy for Low Risk Early Breast Cancer) trial is a Phase III randomized trial evaluating the omission of breast radiation in ER+/Her-2 negative early stage breast cancers with PAM-50 ROR < 60 in women ≥ 50 yo [79]. Until the results of these prospective clinical trials become available, adjuvant breast radiation remains the standard of care after breast conservation surgery for most patients.

## 6. The Potential for Predictive Genomic Assays to Guide Adjuvant Radiation Therapy 

**Figure 1:**
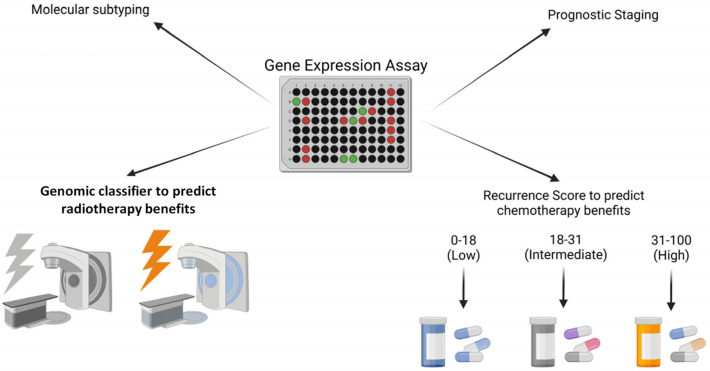
Schematic illustrating the use of a generic genomic assay to help with the process of staging and subtyping breast cancer but also with the ability to provide predictive information with regard to radiation therapy and chemotherapy.

While multiple studies have successfully shown that prognostic genomic assays are able to predict local recurrence in breast cancer [37,38,39], predictive genomic assays could potentially identify subpopulations of breast cancer that are most or least likely to benefit from radiation therapy (Figure 1). Genomic classifiers capable of predicting response to radiation are still under development. However, data has shown that the radiosensitivity of breast cancers appears to be independent of intrinsic molecular subtype and the signaling mechanisms associated with radiosensitivity or resistance may differ among breast cancer subtypes [29,38,80,81].

Speers et al. studied the intrinsic radiosensitivity of 16 human breast cancer cell lines using clonogenic survival assays to develop a 51 gene molecular signature that would identify women at an increased risk of local recurrence most likely to benefit from radiation [82]. This gene classifier was enriched with genes associated with cell cycle arrest and DNA damage response. Moreover, Nimeus-Malmstrom et al. found a reliable gene expression profile capable of predicting local recurrence despite radiation after breast conservation surgery in ER+ breast cancers [83]. Another study identified a 7 gene profile for node positive patients from the Danish Breast Cancer Cooperative Group (DBCG82bc) treated with systemic treatment and randomized to receive or not to receive post-mastectomy radiation therapy (PMRT). This 6 gene expression panel identified patients with “high LRR risk” in which PMRT significantly reduced the risk of LR and “low LRR risk” group in which PMRT showed no additional reduction in LR rate and the response to radiation was independent of intrinsic molecular subtype [81,84].

The Adjuvant Radiotherapy Intensification Classifier (ARTIC), comprised of 27 genes related to cell proliferation, cell cycle and kinase activity as well as patient age, is a gene classifier for radiation sensitivity in patients with high-risk node negative early stage breast cancers was developed using three publicly available cohorts. The ARTIC classifier was the first predictive assay to be later validated with a Phase III clinical trial in which patients were randomized to radiation [85].

The RSI radiosensitivity index is a linear model based on 10 genes (related to DNA damage response, histone deacetylation, cell cycle, apoptosis and proliferation) associated with the fraction of cells surviving after 2 Gy (SF2) of radiation. The RSI has been validated to predict clinical outcomes and benefit from radiation therapy among several cancer types, including breast cancer [86,87,88,89,90]. Torres-Roca et al. [88] later found that RSI may not predict radiation sensitivity among all breast cancer subtypes and may be influenced by ER status. Scott et al. later combined RSI with the linear quadratic model to create the Genome Adjusted Radiation Dose (GARD), a tool believed to predict a tumor’s response to a particular dose of radiation based on its inherent radiosensitivity [86,91].

Other groups have found genomic classifiers for radiation sensitivity that depend on the immune microenvironment. Shen et al. developed an 11 gene signature that stratify tumors based on response to radiation therapy and the tumor immune microenvironment. In this study, CD4+ T cells and B cells infiltrated the radiosensitive tumors and macrophages infiltrated the radioresistant tumors [92]. Cui et al. [93] also developed gene expression signatures based on intrinsic radiosensitivity and antitumor immunity. Validation of these predictive genomic classifiers in prospective clinical trials are warranted before implementation in the clinic.

In summary, while confirmatory studies are needed, many of these pre-clinical studies have identified genes involved in cell proliferation, DNA damage response, immune microenvironment and wound response signaling to be associated with radiation resistance in breast cancer. The results of these studies have been summarized in Table 3.

## 7. Summary and Conclusions

Clinical trials have shown that even the most low-risk breast cancers defined by clinical and pathologic factors alone demonstrate even a modest local control benefit from radiation. Therefore, current treatment guidelines recommend radiation therapy for nearly all breast cancer patients after breast conservation surgery. However, genomic assays are believed to better risk stratify breast cancers based on their inherent biology. While genomic assays for guiding adjuvant systemic therapy decisions in the clinic have been implemented over the past decade, the use of genomic assays in guiding radiation therapy decisions has yet to be adopted. 

Retrospective studies have shown the ability of prognostic genomic assays, such as Oncotype and PAM-50, to predict risk of local recurrence among breast cancer subtypes. These data show promise for identifying low risk breast cancers in which radiation may be safely omitted. Several prospective clinical trials that use Oncotype and PAM-50 genomic assays to guide adjuvant radiation therapy decisions are currently underway and their results are eagerly awaited. Until these clinical trial results are available, post-lumpectomy radiation is recommended for all breast cancer patients < 70 years old regardless of genomic assay results. 

While prognostic genomic assays may help stratify breast cancers according to their risk of local recurrence, the development of predictive genomic assays may help stratify breast cancers according to their likelihood to respond to radiation therapy. The use of predictive genomic assays may one day allow for a more tailored locoregional approach to improve radiation outcomes, however these assays are still under development and there is no consensus on the genes predominantly involved in radiation resistance. For example, lower doses of radiation could be considered in more radiosensitive breast cancer subtypes. In contrast, aggressive radioresistant breast cancers may still experience local recurrence despite radiation therapy. These patients may require more intensive locoregional therapies, such as mastectomy or an increase in radiation dose with tumor bed boost. Currently, the decision to boost the tumor bed is based on clinical factors such as patient age and tumor grade [94], however if a genomic assay were to suggest a more radioresistant breast cancer, radiation dose intensification with tumor bed boost may be considered. In cases of more radioresistant breast cancers, the use of chemotherapies and radiosensitizers may also be considered.

## Figures and Tables

**Table 1:** Prognostic genomic assays in breast cancer.

Microarray	Number of Genes	Genes	Classification	Reference
OncotypeDx	21 genes	16 cancer-related genes associated with proliferation, invasion, and estrogen signaling as well as 5 reference genes	Generates “Recurrence Score” (RS), from 0–100 which stratifies patients into low (0–18), intermediate (18–31), and high (31–100) risk of distant reccurence.	[43]
Mammoprint	70 genes	Genes associated with cell cycle, invasion, metastases and signal transduction	Classifies breast cancer patients into “poor prognosis” or “good prognosis”. Calculates MammaPrint Index (MPI) to stratify Low or High risk of reccurence.	[54,55]
PAM 50	50 genes	50 discriminator genes + 8 controls. These genes identify the intrinsic breast cancer subtypes by indentifying the underlying biology associated with ER and HER2 pathways, and proliferation genes and markers of the basal phenotype.	Calculates a “risk-of-recurrence” (ROR) score, 0–100 which classifies low (0–40), intermediate (41–60), or high (61–100) risk for node-negative cancers.	[68,69,70]

**Table 2:** Current prospective clinical trials evaluating whether prognostic gene assays can identify low risk ER+ early breast cancer patients in which radiation therapy can be safely omitted.

Prospective Clinical Trials	Enrollment Criteria	Aims	Reference
LUMINA	Grade 1–2 invasive breast cancers of luminal A subtype (defined as: ER ≥ 1%, PR > 20%, HER2 negative and Ki67 ≤ 13.25%) in women > 55 yo	To evaluate patients who have low risk of local reccurence following breast conservation surgery and endocrine therapy alone who may be candidates for radiation omission.	[26,27]
IDEA	ER+ invasive breast cancers with RS ≤ 18 who plan to receive endocrine therapy in post-menopausal women	To evaluate locoregional recurrence rates in low risk women via considering tumor biology including OncotypeDx, in which radiation therapy can be omitted after breast conservation surgery.	[71]
PRECISION	ER+ early stage breast cancers < 2 cm with a low risk PAM-50-based ROR in women > 50 yo	To evaluate locoregional recurrence rates in patients omitting radiation treatment after lumpectomy.	[72]
DEBRA	ER+/Her-2 negative early stage breast cancers with RS ≤ 18 in women age 50–69 yo	To evaluate omission of breast radiation after breast conservation surgery and endocrine therapy.	[73]
EXPERT	ER+/Her-2 negative early stage breast cancers with PAM-50 ROR < 60 in women ≥ 50 yo	To evaluate the omission of breast radiation therapy compared to obseravation after breast conserving surgery and endocrine therapy.	[75]
TAILOR-RT	ER+/Her2-negative biomarker low risk breast cancers (defined as RS ≤ 25) with low lymph node burden (1–3+ lymph nodes)	To evaluate the effects on low risk breast cancer patients after ommission or treatment of regional nodal radiation.	[74]

**Table 3:** Summary of gene expression profiles found to be associated with radiation resistance in breast cancer.

Microarray	Number of Genes	Genes	Classification	Reference
Speers et al.	51 genes	Enriched with genes associated with cell cycle arrest and DNA damage response	Identify women at an increased risk of local recurrence most likely to benefit from radiation	[78]
Danish Breast Cancer Cooperative Group (DBCG82bc)	7 genes	HLA-DQA, RGS1, DNALI1, hCG2023290, IGKC, OR8G2, and ADH1B. Genes involving immune system, protein signalling, and metabolism enzymes	Identified patients with “high LRR risk” in which PMRT significantly reduced the risk of LR and “low LRR risk” group in which PMRT showed no additional reduction in LR rate and the response to radiation was independent of intrinsic molecular subtype	[77,80]
Adjuvant Radiotherapy Intensification Classifier (ARTIC)	27 genes	Genes related to cell proliferation, cell cycle and kinase activity as well as patient age	A gene classifier for radiation sensitivity in patients with high-risk node negative early stage breast cancers was developed using three publicly available cohorts	[81]
RSI radiosensitivity index	10 genes	Related to DNA damage response, histone deacetylation, cell cycle, apoptosis and proliferation	Predict clinical outcomes and benefit from radiation therapy among several cancer types, including breast cancer	[82,83,84,85,86]
Shen et al.	11 genes	Genes that look at the tumor immune micro-environment	Stratify tumors based on response to radiation therapy and the tumor immune microenvironment	[88]
Cui et al	33 radiation-related genes	Tumor microenvironment genes such as tumor-associated antigens on the major histocompatibility complex (MHC) molecules.	Stratification of patients by predicting benefits from radiotherapy.	[89]

## Data Availability

Not applicable.
